# Her Heart's Mistake

**Published:** 1889-04-13

**Authors:** E. D. Cumming

**Affiliations:** Author of "An Ordeal by Silence"


### HER HEART'S MISTAKE.

By E. D. Cumming, Author of "An Ordeal by Silence."

*(Continued from page 15.)*

### Chapter I.—*(Continued.)*

Captain Bairdsley made some reply, and turned to Kate. Mrs. Brent's stock of conversation, never very large, was exhausted for the nonce, and she was glad to be relieved of the task of entertaining the new arrival. He, on his side, was quite willing to improve his acquaintance with Miss Harrison, who struck him as the most healthy, animated girl he had seen in Meerut. He, as everyone does, had noticed the pallid, complexionless faces of Anglo-Indian ladies, and the doctor's daughter, though pale, contrasted favourably with them. She was tall and graceful, and about her beauty men had but one opinion, though detractors of her own sex were wont to ask each other how a girl with a mouth like hers could be considered handsome. It was a little too large for strict classical beauty perhaps, but it was easy to forgive a defect which marked such character as Kate Harrison possessed. For the rest, she had fair hair coiled upon a well-shaped head, and brown eyes with which a flirt could have done boundless mischief among the hearts of men.

Captain Bairdsley, to whom Kate had already been pointed out as the belle of the station, applied himself to the congenial task of winning her good opinion, and it is only due to him to say that he did so with well-bred deference and tact. They discovered that they had a good deal in common. Miss Harrison was a thorough musician, and admitted that she sang a little; Captain Bairdsley was the possessor of a good tenor voice, and had brought out any quantity of "pieces" with him, both solos and duets. There was not another man in Meerut just now who could sing a note, and Kate did not disguise her pleasure at learning of his accomplishment. Then it appeared that the Captain was a keen horseman, and had already been looking at one or two horses which the owners were anxious to sell. Kate had her own Arab mare, and rode regularly every morning, either alone or with friends. She knew all the horses and ponies in the station, and was able to advise Captain Bairdsley where to look for the animals he wanted—advice which that gentleman received with becoming gratitude. In due course it appeared that he loved waltzing, and had won a tennis tournament in the best set at Portsmouth. Altogether, when Dr. Harrison came to take his sister and daughter home to dinner at half-past seven, Kate had come to the conclusion that the "new man" was a real acquisition to the station society. While Captain Bairdsley strolled over to his bungalow to change for mess, resolved that one of his first duty calls should be upon that pretty Miss Harrison.

### Chapter II. A Moonlight Picnic.

Captain Bairdsley, attired in grass slippers and the garments known as "pyjamas," was seated in the midst of chaos superintending the unpacking of his baggage, and interviewing the score or two of natives who, having heard of the arrival of a new sahib without retainers, had hastened to apply for the honour of serving him. Vultures are not quicker to espy carrion than are Indian servants to discover a possible master, and the verandah of the little bungalow which Captain Bairdsley had arranged to share with a brother officer, Captain Henry Phelps, was crowded with the "unemployed." Every man bore a handful of well-thumbed "chitties," or characters, bearing testimony to his personal merits and professional talents as khitmugar, bearer, or syce, as the case might be.

The Captain's feeling on reading the first applicant's "chitties" was one of surprise that a menial of such incomparable honesty, cleanliness, and wisdom should be out of work at all. He was about to appoint him his bearer on the spot, when it occurred to him to examine the dates of the testimonials, which were almost illegible in their rich coating of oil and dirt. The result was unsatisfactory. H. Baynes, of the 150th, certified that Gopal Singh had been his bearer at Morar from May, 1879, until the end of August, 1882, during which time the said Gopal

Singh had given complete satisfaction. So far so good. But the next chit, signed by William Simpson, of the Bengal Civil Service, certified that Gopal Singh had acted as *his* bearer at Allahabad from June, 1880, until January, 1884, during which time he had never once failed in his duties in the smallest degree. There was a discrepancy somewhere, and Captain Bairdsley solved the difficulty by returning the man his precious papers, and telling him to "jao"—this useful monosyllable being one of the first words of Hindustani acquired by new arrivals. Gopal Singh made no protest; he salaamed with all humility, and went away to return to their proper owners the chitties he had temporarily hired, cursing his own folly in not showing them to the "petition writer" in the bazaar before he took them to the sahib. The simple-minded Aryan had no idea how he had been found out, and Captain Bairdsley's penetration earned for him a high place in Gopal Singh's respect.

"Well, Bairdsley," said Captain Phelps, coming into the room, and wiping the moisture from his brow, "suited yourself with niggers yet? Can I help you?"

"You're very good, Phelps. If you would tell your bearer to pick out one or two of the likeliest men from the crowd outside, and put them through their facingss, I should be immensely obliged to you."

"I saw a fellow among them who used to be with Wilton of ours, a few months ago. Good servant he was, too. I'll tell my man to begin with him."

Captain Phelps' servant received his instructions, and in a quarter of an hour George Bairdsley found himself the master of a bearer to look after his clothes and dress him, and a khitmugar, whose business would be to wait on him at table. And that being settled, the new domestics entered upon their duties at once, being apparently unembarrassed by luggage of any description.

"They will bring their property some time or other—if they've got any," said Captain Phelps, in answer to his friend's remark. "You need not trouble yourself about them. They will soon let you hear of it if they don't like their place."

"What is the recognised time for making calls in this country, Phelps? I suppose I must go and pay my respects all round the station."

"Have you got any letters of introduction?"

"No."

"Then you'd better begin with the civilians, and work your way down. Ladies are always 'at home' between twelve and two—the hottest hours of the day. It's barbarous cruelty to the caller, but it has to be done."

"I can wear whites, I suppose?"

"O yes; they are merciful on that score. I'll send for a gharry for you, and make out a list of the people, while you're changing."

"Many thanks," and Captain Bairdsley waded through the mass of crumpled papers, which littered the floor round his packing cases, to his own bedroom, where with his bearer's silent aid he dressed himself in a suit of white drill.

It was not a very formidable list that Captain Phelps handed him when he emerged from his apartment. There were only a dozen or fifteen names upon it, and all the houses were tolerably close together.

"One advantage—the only advantage—you cut in for by coming at this time of the year is that most of the mem-sahibs are away up in the hills," said his friend; "but there are quite enough left to give you two days' work. You'd better start at once, as it's nearly twelve now, and the gharry has come."

So Captain Bairdsley climbed into the rattling gharry, after a deprecating glance at the attenuated horse and its rope-patched harness; and, with his bearer on the footboard behind to direct the driver, started to pay the round of formal visits with which a new-comer is expected to inaugurate his appearance at an Indian station.

First to the Collector's beautiful bungalow, which was twice the size and height of any other in Meerut. Mrs. Macdonald was at Mussoorie, and Mr. Macdonald, the collector, was engaged, and so Bairdsley "shot his pasteboards," to use the Anglo-Indian slang, and took the next house, the General's, whence he escaped with equal ease. Then duty prompted him to call upon Mrs. Tenterdale, his colonel's wife, with whom he spent twenty minutes, discussing his passage out, the heat, the scarcity of rideable horses, and the iniquities of native domestics; and then, having confessed where he had called that day, was confidentially recommended to go next to Mrs. Strachan, the Deputy-Collector's wife, "who is a little sensitive about precedence, Captain Bairdsley, as those civilians often are."

Captain Bairdsley expressed his thanks for the hint, and, after shouting "Strachan sahib's!" at his bearer with unnecessary fierceness, got into the gharry and mopped the perspiration from his face and hands. The vehicle lumbered out on to the Mall again in the overpowering heat and glare, and the occupant looked through the broken venetians which replace glass windows in Indian hackney carriages, wondering what had become of the population. The only human creature in sight was a native orderly on his camel, shambling along amid a cloud of dust, evidently on some errand. A few scraggy goats lay about on the roadside, but other living beings there were none.

The gharry turned off the Mall up a narrow bye-road, and as it did so Captain Bairdsley caught sight of the name on the gate of the corner bungalow. "Whoa!" he shouted, rightly supposing that he would be understood. As he was passing the Harrison's house, he might as well go in, though the doctor's was by no means the next name upon Captain Phelps' list.

"Go in there," he said to the bearer, who had scrambled off his perch, and stood awaiting his master's commands at the gharry door; "that house" (indicating it with his stick). If he carried out Mrs. Tenterdale's directions, and went to call first upon the exacting Mrs. Strachan, he might not get away in time to see Miss Harrison that day; and the prospect of including her in his round of duty calls was one which did something towards reconciling him to the whole task.

He soon found himself in the darkened drawing-room, and noted that the moment he entered it the punkah began to wave, and the thermantidote commenced its rapid, even sobbing behind the water-drenched kuskus tattie which filled the doorway to the back verandah.

"A well-managed house," said Captain Bairdsley to himself. "Mrs. Tenterdale had to call a dozen times before anyone pulled her punkah, and she hadn't a thermantidote at all. I wonder who has charge of the establishment; the old lady, I suppose."

The room was deliciously cool, and restful to the eye after the heat and dazzle of the dusty road, and Kate, in a loosely-made flowing gown of white, harmonised well with her surroundings when she came in to receive him.

"I have to ask you to excuse my aunt's non-appearance, Captain Bairdsley," she said, as they shook hands; "she feels the heat so much that she makes a practice of lying down at this time of the day."

The Captain made the necessary reply, and they seated themselves for a chat.

"I must warn you not to sit near the thermantidote if you are at all liable to rheumatism," said Kate; "the cold, damp draught through the kuskus is a certain way of bringing it on."

But Captain Bairdsley was not liable to rheumatism, and said so, thinking in his heart that this was one of the most sensible girls of her age he had ever met. She could not be more than eighteen or nineteen at most, and she had the frank manner and self-possession of an experienced hostess.

It was surprising how much they found to say to each other, in view of the fact that they only met for the first time the day before. The visitor forgot to look at his watch, and Mrs. Tenterdale's hint regarding the Deputy-Collector's wife's touchiness on points of "precedence" faded from his mind as he lounged in a comfortable chair talking to Miss Harrison and listening to the music of her voice. There was fascination in the twilight of the room and her low, sweet tones; and when a pause in their conversation reminded him that he had made a rather lengthy call, he was astonished to find that it was quarter past two. He rose to take his departure, half thankful that the hour forbade his continuing his journey, and that Mrs. Strachan must wait until another day. After all, what did it matter? It was only natural that he should take each house as it came in his way, and no one was likely to ask where he had called and where he had not called. But Captain Bairdsley reckoned without his host as he drove back to his own quarters thinking these things.

"How do you like Captain Bairdsley?" asked Mrs. Tenterdale of Mrs. Strachan that evening at the club.

"He has not called yet," answered Mrs. Strachan, "but as he only arrived yesterday, perhaps it's rather soon to expect him."

"Has not called?" echoed the Colonel's wife. "He came to see me to-day, and said he was going to you next."

"Well, he never turned up," said Mrs. Strachan a little snappishly. "I suppose he went somewhere else instead."

"Very remiss of him if he did," rejoined Mrs. Tenterdale. "The man must know whom he ought to visit first."

"I saw a gharry go into the Harrison's to-day shortly before one," said Mrs. Pringle, the wife of the only English lawyer in Meerut. "I heard a European shouting and peeped out, thinking it might be a visitor for me, and I was not ready to receive anyone. Whoever it was stayed there until after two, and drove up the Mall towards the 110th mess-house afterwards."

"Captain Bairdsley is sharing Captain Phelps' bungalow, close to their mess," said Mrs. Tenterdale to no one in particular.

And then there was silence for a minute or two, while fans fluttered lazily.

"Well, I suppose we must excuse Captain Bairdsley's ignorance of Indian ways," said Mrs. Strachan, with somewhat acid magnanimity; "but really, my dear Mrs. Tenterdale, an hour and a half is a very long time for a first call."

"Very," assented that lady. "I think that Mrs. Brent generally lies down during the day, does she not?"

"I believe so," answered Mrs. Strachan, "she told me the other day that she never saw visitors in the hot weather."

"Hem! Then we mustn't be hard upon Captain Bairdsley, perhaps," said Mrs. Tenterdale.

So the Captain's neglect of social duty was pardoned in consideration of the interesting conjectures to which his long visit at the corner bungalow had given rise; and when he made his appearance at Mrs. Strachan's on the following day he was received with all that lady's usual graciousness.

"Have you heard of this perfectly inspired idea of Mrs. Pringle's, Kate?" enquired Mrs. Arkwright of the doctor's daughter, a few evenings afterwards, as they sat by the tennis court watching the players.

"Her moonlight picnic to the Acacias? Yes, I was discussing it with her to-day."

"It ought to be glorious fun," said Mrs. Arkwright."

"Everyone in the station has been asked," said Mr. Wentworth, who was, as usual, hovering about Miss Harrison; "all our fellows are going, except Major Weekes and the fellows on duty. May I ride out with you, Miss Harrison?" he asked, with a vivid blush.

"Oh, yes; you shall escort me, Mr. Wentworth," replied Kate, with her brightest smile. "You will have to give your pony a rest that day, though, as the Acacias bungalow is eight miles out."

"I will," said the youth, whose ninety-rupee tat covered more ground every day than any other animal in Meerut. "You wo'n't forget, Miss Harrison?" he added, anxiously.

"Oh, no; I wo'n't forget," answered Kate; and the blushing Tops went away happy.

But when the eventful evening came round, and the picnic party assembled in Mrs. Pringle's compound at nine o'clock, poor Tops found that he was doomed to disappointment. Captain Bairdsley, who had not yet succeeded in purchasing a horse, had been mounted for the occasion by Dr. Harrison, and, of course, it was only natural that Miss Harrison would like to see how the sturdy little country-bred cob carried him. Still, it was surely unnecessary for the Captain to remain at Kate's girths so exclusively, and monopolise her conversation to the extent he did. Mr. Wentworth rode along the smooth unmetalled road beside his divinity, feeling that he was a superfluity, and was aggrieved at Kate's neglect of her promise. She did not attempt to send him away, it was true, but neither did she make the smallest effort to dismiss Captain Bairdsley; and when he suggested that they might "trot on a bit," by way of giving her a hint, the Captain trotted on with them. There was no getting rid of him, and, to make matters worse, Miss Harrison did not seem the least anxious to do so. He was beginning to realise the truth of that vulgar adage about "two" and "three," when Mrs. Tenterdale cantered up alone, and joined them. She had a word for Captain Bairdsley, and another for Kate, and presently turned to Mr. Wentworth:

"My horse is so fresh to-night," she said. "I must give him another smart canter before he will consent to walk like a reasonable creature. Will you come with me?"

And the diffident Tops, to whom the Colonel's wife's wishes were as much law as the Colonel's orders, was swept away, a miserable subaltern. Mrs. Tenterdale was deservedly popular among the young officers of her husband's regiment, and the honour she to-night conferred upon the youngest had been eagerly sought by many. It is to be feared that her favoured escort did not value the distinction at its proper worth; but, as Mrs. Tenterdale said afterwards to Mrs. Pringle, her elopement with Mr. Wentworth made two people happy at the expense of one. And the two people whose happiness was thus thoughtfully cared for, rode on side by side in the lovely moonlight, now walking, when the overhanging boughs cast deep shadows on the ground, now cantering freely over the soft sandy plain, whose level was unbroken by a shade that might frighten the most timid of horses. Before and behind them were groups and couples of horsemen and horsewomen, laughing and talking with all the high spirits engendered by the coolness of the night. A deep sense of blissful content was creeping over George Bairdsley's soul. He had known Kate Harrison for only one short week, but in that brief space of time he had played tennis with her, accompanied her on her morning ride, danced with her, and knew that he looked forward to meeting the girl more eagerly each day. He had always been a favourite among women, and the beating of more than one heart had quickened at his approach ere now; but his own affections had never been bestowed. He came out to India an independent heart-whole man. He told himself when he embarked that he had drained social pleasures to the very dregs, and that the companionship of ladies palled upon him; that in Meerut he should see nothing which would even remind him of the series of luxury and refinement he had been used to frequent. He had often met Anglo-Indian ladies at home, and the contrast between them and their untravelled sisters had led him to look for a languid, uninteresting circle, doubly unattractive to one who had moved in the best local society in England. And how soon his eyes had been opened! He found refinement as true as that he had left. He found an all-pervading air of the broadest hospitality; a total absence of the veneer which must be pierced and dissolved by long acquaintance before real friends can be made at home. There was a frankness, an open-hearted, genuine friendliness about this little coterie for which he had not been prepared; and, above all, there was Kate Harrison.

Captain Bairdsley's preconceived ideas about the life he was to lead in Meerut had undergone a change, and as he rode at her side in the moonlight he felt that the influence which had caused it was hers. It seemed as though Kate had been reading his thoughts, for after a silence which he felt no wish to break, she touched upon the very subject which filled his mind.

"Tell me, Captain Bairdsley," she said, "is India in the least like the country you expected to find?"

"In what respect, Miss Harrison?" he queried in return.

"It was rather a wide question," laughed Kate. "Take ourselves, for instance. Do not people out here seem quite different to those at home?"

"They are very different, and not in the least what I expected."

"Why, what did you expect?"

"I suppose I must have formed my expectations on the few Anglo-Indians I have seen at home; but I am most agreeably surprised at what I have found."

"I think one gets to know people better out here," said Kate thoughtfully; "we get a closer insight into each other's daily life."

"I already know some people whom I want to make my friends," said Bairdsley.

"I hope you will succeed," answered Miss Harrison.

"I hope so, too," he said meaningly, and relapsed into silence again.

They reached the Acacias bungalow in company with a number of other friends who had ridden up to overtake them as the house came in sight; and as Captain Bairdsley took Kate's hands to help her dismount, she thought he held them a shade longer than was absolutely necessary. The syces, who had been sent on earlier in the day, took the horses away, and the party sat down in the verandah, or wandered about the compound, waiting until supper was ready. Cold meats, pastry, fruit and sweets in profusion formed the meal, and iced drinks of various kinds, temperance and otherwise, were in great demand. Mrs. Pringle's, like all moonlight picnics, was an unconventional entertainment, somewhat boisterous, but thoroughly enjoyable.

*(To be continued.)*

